# The Immunohistochemical Expression of REV-7 in Various Human Cancer Pathology Specimens: A Systematic Review

**DOI:** 10.7759/cureus.52542

**Published:** 2024-01-19

**Authors:** Theodoros Spinos, Dimitrios Goutas, Tatiana S Driva, Eleni Zografos, Charikleia Gakiopoulou, George Agrogiannis, Vasiliki Zolota, Vasiliki Tzelepi, Ioannis Manolis, Efthymios Koniaris, Maria Ioannou, Andreas C Lazaris

**Affiliations:** 1 First Department of Pathology, National and Kapodistrian University of Athens, Athens, GRC; 2 Department of Clinical Therapeutics, Oncology Unit, Alexandra General Hospital, Athens, GRC; 3 Department of Pathology, University Hospital of Patras, Patras, GRC; 4 Department of Pathology, Hippokration General Hospital, Athens, GRC; 5 Department of Pathology, University of Thessaly, Larissa, GRC

**Keywords:** chemoresistance, prognosis, cancer, immunohistochemistry, rev-7

## Abstract

The purpose of this systematic review is to summarize all existing evidence, regarding the immunohistochemical expression of REV-7 in different human cancer pathology specimens. Moreover, the association of REV-7 expression with disease severity (clinical course), patients’ survival, prognosis, and response to various treatments, such as chemotherapy and irradiation, was investigated. Three databases (PubMed, Scopus, and Cochrane) were systematically screened, from inception to September 2, 2023, as suggested by the Preferred Reporting Items for Systematic Reviews and Meta-Analyses (PRISMA) statement. Only studies using immunohistochemical staining for REV-7 in paraffin-embedded cancer tissues were included. Nine studies met the inclusion criteria and were included in the final qualitative synthesis. All nine studies were retrospective and non-comparative ones. Selected studies reported immunohistochemical expression of REV-7 in different types of cancer, including testicular cancer, ovarian cancer, esophagus squamous cell carcinoma, prostate cancer, colorectal cancer, diffuse large B-cell lymphoma, breast cancer, lung cancer, and skin cancer. High REV-7 expression was associated with faster disease progression, resistance to available treatment options, and worse prognosis in the majority of included studies. These results indicate that immunohistochemical staining of REV-7 protein could potentially be used as a predictive tissue marker in certain cases. Promising results, arising from REV-7 inactivation experiments, render REV-7 targeting a potential therapeutic strategy for future cancer management, especially in the cases of chemoresistant or radioresistant disease.

## Introduction and background

REV-7 (also known as REV7, MAD2L2 and MAD2B) represents a subunit of DNA Polymerase ζ (Polζ), which plays a crucial role in skipping DNA-damaged lesions [1]. Many endogenous and exogenous factors can result in DNA damage, including chemotherapy drugs, such as cisplatin. Cells have developed several mechanisms in an effort to cope with all these different factors causing DNA damage, which all together form the DNA damage tolerance system. All mechanisms of the DNA damage tolerance system work together in cooperation to ensure the completion of DNA replication [2]. One of these mechanisms is the translesion DNA synthesis (TLS), while the Polζ represents one of its most important ingredients [3-5]. REV-7 and REV-3, which are in turn another protein subunit of Polζ, bind together in order to form the Polζ. In that complex, REV-3 has a catalytic action, functioning as a polymerase, while REV-7 has an accessory action, enhancing the polymerase activity of REV-3 [6]. REV-7 was first found in the yeast *Saccharomyces cerevisiae* (*S. cerevisiae*), playing a crucial role in the implementation of the misrepair mutagenesis [7,8]. Beyond its significance in TLS and cell cycle checkpoint, REV-7 has been found to be engaged in several other important biological procedures, such as homologous recombination (HR), DNA double-strand break (DSB) repair, preservation of telomeres, cellular transcription and molecular signaling, epigenetic modifications, and survival of primordial germ cells. Recent studies, involving both cell lines and pathology tissues originating from different human cancers, reported an important association of REV-7 protein with cancer pathogenesis, development and spreading, while it has also been proposed as a useful target for cancer management [9,10]. The purpose of this systematic review is to summarize all existing evidence, regarding the immunohistochemical expression of REV-7 in different human cancer pathology specimens. Moreover, the association of REV-7 expression with disease severity (clinical course), patients’ survival, prognosis and response to various treatments, such as chemotherapy and irradiation, was investigated.

## Review

Materials and methods

Search Strategy

Three databases (PubMed, Scopus and Cochrane) were systematically screened, from inception to September 2nd, 2023, as suggested by the Preferred Reporting Items for Systematic Reviews and Meta-Analyses (PRISMA) Guidelines. The reference section of included studies was also investigated for finding other possibly acceptable studies. Only human studies and articles in English were accepted. The following search string was used: (rev7 OR rev-7) AND (immunohistochemistry OR immunohistochemical OR pathology). Two of the authors (T.S. and D.G.) independently reviewed the three databases, while disagreements between the two authors were resolved by a third reviewer (A.L.), until a final decision was made.

Selection Criteria and Data Extraction

Selection of included studies was based on the PICO (Patients, Intervention, Comparison, Outcome) criteria. Patients (P) should be adults (>18 years old) diagnosed with different types of cancer. The Intervention (I) should be REV-7 immunohistochemical staining of cancer tissues, which were either retrieved after surgical excision or biopsy of the lesion. Comparison (C) was made between REV-7 expression in target cancer tissues and/or adjacent normal tissues and/or other cancer tissues originating from human patients. However, simple observational studies and case series with no comparison group were also included. The Primary Outcome (O) was the immunohistochemical expression of REV-7 in target cancer tissues. Both the quantity (low versus high) and the quality (cytoplasm versus nucleus) of this expression were studied. The Secondary Outcomes (O) were the association of REV-7 expression with disease severity, patients’ survival and prognosis and response to various treatments, such as chemotherapy and radiation. Comparative prospective and retrospective studies were eligible. Studies evaluating the immunohistochemical expression of REV-7 in different cancer types, in general, were also eligible. Non-comparative studies and case series were included. Ex vivo studies and articles including only cell lines, without human tissues and pathology specimens, were excluded. Articles including only molecular approaches, such as polymerase chain reaction (PCR), next-generation sequencing (NGS) and microRNAs (miRNAs), fluorescence in situ hybridization (FISH) and enzyme-linked immunosorbent array (ELISA), without reporting the use of immunohistochemical staining in paraffin-embedded cancer tissues, were not included.

Article Selection

**Figure 1:**
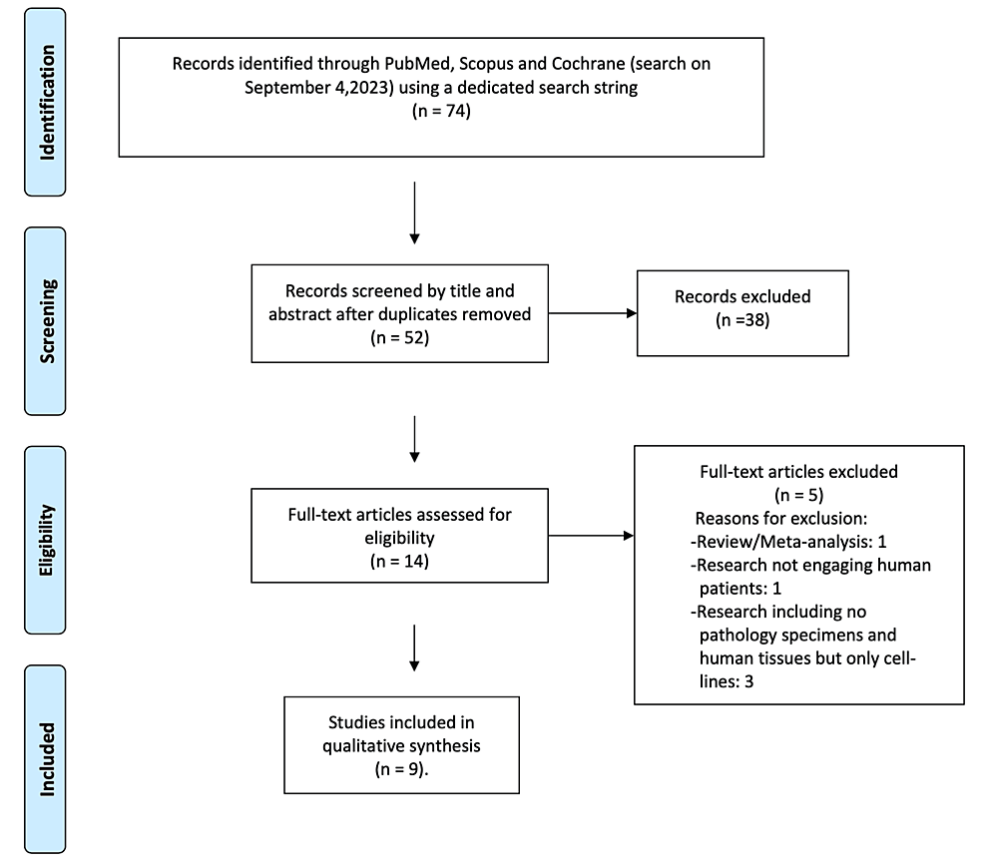
The Preferred Reporting Items for Systematic Reviews and Meta-Analyses (PRISMA) flow chart for the selection of included studies.

The initial search strategy retrieved 74 articles (PubMed: 44, Scopus: 30, Cochrane: 0). When duplicates were removed (n=22), 52 remaining studies were screened. Thirty-eight of these studies were excluded after reading the title and the abstract, while 14 full-text articles were evaluated for eligibility. Subsequently, the SQR3 (Survey, Question, Read, Recite, and Review) technique was used and nine relevant articles were selected and included in the qualitative synthesis, while five were excluded. One study was excluded because it was a review article, one study was excluded because the research did not involve human patients and three studies were excluded because they reported experiments that were performed solely on cell lines (n=3). The PRISMA flow chart for the selection of included studies is presented in Figure 1.

Results

Characteristics of Studies

The characteristics of the included studies are presented in Table 1. In total, nine studies were included in the final qualitative synthesis [11-19]. The oldest study was published in 2014 [12], while the most recent one was published in 2021 [19]. The number of pathology specimens or human cancer tissue samples which were included in selected studies ranged from 21 [17] to 188 [19]. All studies were retrospective and non-comparative [11-19]. Two studies reported using an anti-REV-7 antibody which was produced by Abcam® (Cambridge, UK) [13,18], another study reported using an anti-MAD2L2 antibody which was produced by Proteintech® (Wuhan, China) [15], another research team utilized an anti-MAD2B (REV7) antibody from BD Biosciences® (Franklin Lake, NJ, USA) [11], while three studies employed a Rabbit Polyclonal Anti-REV7 antibody [12,16,19]. Finally, only a single study did not provide any details about the antibody which was used for the evaluation of REV-7 expression in paraffin-embedded cancer tissues [17]. Interestingly, all nine selected articles reported immunohistochemical expression of REV-7 in different types of cancer, including testicular cancer [11], ovarian cancer [12], esophagus squamous cell carcinoma (SCC) [13], prostate cancer [14], colorectal cancer [15], diffuse large B-cell lymphoma [16], breast cancer [17], lung cancer [18] and skin cancer [19]. All nine studies were classified as Level 4, according to Levels of Evidence for Therapeutic Studies (from the Centre for Evidence-Based Medicine, http://www.cebm.net). For practical reasons, we further divided the Results section of this paper according to the affected body system or the corresponding medical specialty involved. For certain types of cancer, such as breast and skin cancer, there is an overlap of medical specialties treating the same condition. Selected studies can be further divided into the following sections: REV-7 in Urology (testicular and prostate cancer) [11,14], REV-7 in Gynecology (ovarian and breast cancer) [12,17], REV-7 in Gastrointestinal Tract (esophagus and colorectal cancer) [13,15], REV-7 in Hematology (lymphoma) [16], REV-7 in Respiratory Tract (lung cancer) [18] and REV-7 in Skin (skin cancer) [19].

**Table 1:** Characteristics of included studies *Levels of Evidence for Therapeutic Studies (from the Centre for Evidence-Based Medicine, http://www.cebm.net).

Study Name	Journal	Type of Cancer	Number of Pathology Specimens	Antibody	Type of Study	Level of Evidence*
Sakurai et al. [11]	Cancer Letters (2020)	Testicular Cancer (seminoma, embryonal carcinoma, yolk sac tumor, teratoma, malignant lymphoma)	78	Anti-MAD2B (REV7) antibody (BD Biosciences, Franklin Lake, NJ, USA)	Retrospective	4
Niimi et al. [12]	Cancer Science (2014)	Ovarian Cancer (serous adenocarcinoma, mucinous adenocarcinoma, endometrioid adenocarcinoma, clear cell carcinoma)	137	Rabbit Polyclonal Anti-REV7 antibody	Retrospective	4
Gu et al. [13]	Cancer Science (2019)	Esophagus Squamous Cell Carcinoma	102	Anti-REV7 antibody (Abcam, Cambridge, UK) at a dilution of 1:100 overnight	Retrospective	4
Kurfurstova et al. [14]​	Molecular Oncology (2016)	Prostate Cancer	103	N/A (Details are not provided)	Retrospective	4
Li et al. [15]​​​​	Molecular Oncology (2018)	Colorectal Cancer	100	Anti-MAD2L2 (Proteintech, Wuhan, China)	Retrospective	4
Okina et al. [16]	International Journal of Hematology (2015)	Diffuse Large B-Cell Lymphoma	83	Rabbit polyclonal anti-REV7 antibody	Retrospective	4
Feng et al. [17]​​​​​​​	Oncology Research (2016)	Breast Cancer	21	N/A (Details are not provided)	Retrospective	4
Sanoyama et al. [18]​​​​​​​	Pathology International (2020)	Lung Cancer (small cell lung carcinoma, adenocarcinoma, squamous cell carcinoma, large cell neuroendocrine carcinoma)	184	Anti‐REV7 antibody (ab180579, rabbit monoclonal; Abcam, Cambridge, UK)	Retrospective	4
Hoshino et al. [19]​​​​​​​	Pathology International (2021)	Skin Cancer (malignant melanoma, squamous cell carcinoma and basal cell carcinoma)	188	Rabbit Polyclonal Anti-REV7 antibody	Retrospective	4

REV-7 in Urology

Sakurai et al. studied the immunohistochemical expression of REV-7 in 78 testicular cancer tissue samples [11]. Out of these tissue samples, 53 were seminomas, 11 were embryonal carcinomas, three were yolk sac tumors, eight were teratomas, and finally, three were malignant lymphomas. Evaluating REV-7 intensity scores (IS) based on expression in the nuclei, they used the following scores: 0 (no expression), 1 (weaker expression than germ cells in normal seminiferous tubules), 2 (equal expression to normal seminiferous tubules) and 3 (stronger expression than normal seminiferous tubules). Regarding the normal adjacent tissues (non-cancer tissues), the authors reported REV-7 expression in germ cells in the seminiferous tubules, mainly in the nuclei. As far as testicular cancer tissues are concerned, 72 out of 78 samples tested positive for REV-7 expression. Interestingly, all the seminomas, the embryonal carcinomas and the yolk sac tumors were REV-7 positive. On the contrary, only four out of the eight included teratomas and one out of the three malignant testicular lymphomas tested positive for REV-7 expression. They concluded that testicular germ cell tumors are associated with a high REV-7 immunohistochemical expression [11].

Kurfurstova et al. investigated the immunohistochemical expression of several proteins, including REV-7, in 103 patients with sporadic prostate cancer who underwent radical prostatectomy [14]. The following IS was used: 0 (negative expression), 1 (weak expression), 2 (moderate expression) and 3 (strong expression). Most importantly, the authors chose for each patient three blocks, consisting predominantly of benign prostatic hyperplastic (BPH) tissue, prostate intraepithelial neoplasia (PIN) tissue and prostate cancer tissue, respectively. Thus, they were able to evaluate different immunohistochemical expressions of target proteins, including REV-7, during disease progression. The authors reported the reduction of REV-7 expression during disease progression from BPH, through PIN, to prostate cancer. Moreover, they reported that in contrast to the other proteins which were studied, the decreased expression of which was only evidenced in small patches of target tissues as the disease progressed, loss of REV-7 expression was more extensive, being reported in large areas of prostatic tissues [14].

REV-7 in Gynecology

Niimi et al. studied the immunohistochemical expression of REV-7 protein in 137 ovarian cancer tissue samples [12]. Out of these tumors, 47 were serous adenocarcinomas, 19 were mucinous adenocarcinomas, 22 were endometrioid adenocarcinomas and 49 were clear cell carcinomas. The IS of REV-7 staining was scored as follows: 0 (negative expression), 1 (weak expression), 2 (medium expression) and 3 (strong expression). It was further divided into two large categories: low expression (scores 0 and 1) and high expression (scores 2 and 3). REV-7 was found to be expressed predominantly in the nuclei of cancer cells and not in the adjacent non-cancer cells. In total, 126 out of 137 tissue samples tested positive for REV-7 immunohistochemical expression, 54 of which were characterized by low REV-7 expression and 83 by high REV-7 expression. Interestingly, all 49 clear cell carcinoma tissue cases tested positive for REV-7 expression (13 of which exhibited low REV-7 expression and 36 high REV-7 expression), while only 77 out of 88 non-clear cell carcinoma tissues tested positive for REV-7 (41 of which were low REV-7 ones and 47 of which were high REV-7 ones). They concluded that the clear cell carcinoma histological type is associated with high REV-7 expression, in a statistically significant manner. Finally, they correlated REV-7 expression with prognosis, reporting that high REV-7 expression was significantly associated with worse progression-free survival (PFS) in advanced-stage disease cases [14].

Feng et al. investigated the significance of REV-7 expression in breast malignancies [17]. For this purpose, 15 normal breast tissue samples and 21 breast cancer tissue samples were acquired. According to their findings, REV-7 was more highly expressed in breast cancer tissues when compared with normal breast tissues. Moreover, REV-7 was found to be highly expressed in the majority of the high-grade breast cancer tissues. Finally, they reported that higher REV-7 expression in breast cancer tissues was associated with patients’ shorter survival, as opposed to patients whose tissues showed a lower REV-7 expression. They thus concluded that a negative correlation exists between REV-7 expression and survival of breast cancer patients [17].

REV-7 in Gastrointestinal Tract

Gu et al. studied the expression of REV-7 in 102 esophageal SCC tissues and compared them with matched adjacent (52 specimens) or normal tissues (21 specimens) [13]. In their study, immunohistochemical expression of REV-7 was evaluated using a score of 0 to 3 points with intervals of 0.2 points. REV-7 immunohistochemical staining was higher in esophageal SCC tissues (score: 2.2 ± .15) when compared with the adjacent (score: 1.4 ± .11) or normal (score: 0.8 ± .17) tissues, in a statistically significant manner. As expected, REV-7 immunohistochemical expression was pronounced in the nuclei of cancer cells. Therefore, researchers arrived at the conclusion that an elevated REV-7 immunohistochemical expression may be a tissue marker of esophageal SCC [13].

Li et al. investigated the immunohistochemical expression of two proteins, REV-7 and NCOA3, in colorectal cancer tissues [15]. They reported statistically significant higher expression of both proteins in colorectal cancer tissues, in comparison with the adjacent normal tissues. Interestingly, higher expression of REV-7 was correlated with lower expression of NCOA3, while low REV-7 expression was correlated with high NCOA3 expression. Regarding the association between REV-7 expression and various clinicopathological characteristics, the authors reported that higher REV-7 immunohistochemical expression was associated with smaller tumor volume, earlier TNM stage, less invasion, and a lower possibility of distant metastasis in colorectal cancer patients. They commented that these findings suggest that REV-7 could act as a suppressor of colorectal cancer progression and metastasis. Furthermore, high REV-7 expression was a predictive factor of favorable prognosis, while high NCOA3 expression was associated with poor prognosis. In their cohort, patients with high REV-7 expression and low NCOA3 expression were associated with the best outcomes, whereas patients with low REV-7 expression and high NCOA3 expression were associated with the worst outcomes. Their results imply that REV-7 might possibly impede the development of colorectal cancer [15].

REV-7 in Hematology

Okina et al. studied the expression of REV-7 protein in 83 tissue samples from patients diagnosed with diffuse large B‐cell lymphoma who underwent rituximab and CHOP chemotherapy (R-CHOP) [16]. Fifty-six tissues were of nodal origin and 27 were of extranodal origin. Immunohistochemistry intensity was scored as follows: 0 (no staining), 1 (weak expression), 2 (moderate expression) and 3 (intense expression). A score of 3 was considered as high expression, while scores of 0, 1 and 2 were considered as low expression. The average IS for all diffuse large B‐cell lymphoma tissues was 2.04. The authors reported frequent expression of REV-7 in the nuclei of the lymphoma cells, while REV-7 staining was almost absent in adjacent normal lymphocytes and barely detectable in germinal center lymphocytes. However, they found no association between REV-7 expression and various clinicopathologic features, but only a weak correlation with the extranodal location of the lymphoma (p=0,1). Interestingly, high REV-7 expression was associated with significantly lower overall survival (OS) and PFS, while it was also an independent prognostic factor of poor prognosis in diffuse large B‐cell lymphoma patients [16].

REV-7 in Respiratory Tract

Sanoyama et al. reported REV-7 expression in lung cancer tissues [18]. For their study 84 surgically excised lung cancer tissues, including 21 small cell lung carcinomas (SCLCs), 21 adenocarcinomas, 21 SCCs and 21 large cell neuroendocrine carcinomas (LCNECs), along with 100 SCLC biopsy specimens were used. The immunohistochemistry intensity for REV-7 was scored as follows: 0 (negative expression), 1 (weak expression) and 2 (strong expression). Scores of 0 and 1 were considered as low REV-7 expression, while a score of 2 was considered as high REV-7 expression. REV-7 staining was mainly expressed in the nuclei of lung cancer cells, while it was absent in the adjacent non‐cancer cells. Twenty-one out of 21 SCLC tissues tested positive for REV-7 expression (eight specimens had an IS of 1 and 13 specimens had an IS of 2). Seven out of 21 adenocarcinomas tested positive for REV-7 expression and 14 tested negative. Regarding SCC tissues, nine were REV-7 positive and 14 were REV-7 negative, while regarding LCNEC tissues, 14 out of 21 were REV-7 positive and nine were REV-7 negative. They thus concluded that SCLCs had a significantly higher REV-7 expression in comparison with other types of lung cancer. The authors reported that although a significant correlation between REV-7 expression and prognosis was evidenced, the level of REV-7 expression was negatively correlated with extensive-stage disease and the possibility of distant metastasis. Finally, they showed that high REV-7 expression was also associated with higher cancer cell proliferation in SCLCs, as evidenced by the significantly higher Ki-67 Lis in the high REV-7 expression group [18].

REV-7 in Skin

Hoshino et al. reported the expression of REV-7 in 188 skin cancer tissues (96 malignant melanomas, 51 SCCs and 41 basal cell carcinomas) [19]. IS for REV-7 staining was scored as follows: 0 (negative expression), 1 (weak expression) and 2 (high expression). Scores of 0 and 1 were considered as low REV-7 expression, while an IS of 2 was considered as high REV-7 expression. The immunohistochemical analysis revealed that 87 out of 96 melanomas were REV-positive, with only nine being REV-7 negative. The authors also investigated the association of REV-7 expression with cancer cell proliferation, reporting that high REV-7 expression was associated with significantly higher Ki-67 LIs. Moreover, they showed that high REV-7 expression in malignant melanoma tissues was also significantly associated with increased tumor thickness, which represents one of the most important prognostic indexes of skin melanomas. Finally, they reported positive REV-7 expression in the majority of the squamous cell (38 out of 51) and basal cell (34 out of 41) carcinomas. High REV-7 expression was also correlated with cancer cell growth in squamous and basal cell carcinomas, proving that high REV-7 is not only associated with melanoma cell proliferation but also with squamous and basal cell cancer cell proliferation [19].

Discussion

REV-7 is a putative protein actively involved in DNA repair and its complex role in cancer biology has not been fully elucidated. Characteristically, a recently published review study that focuses on the functions of REV-7 protein in human cancer cells reports its usefulness in cancer management and treatment [20]. According to the authors, REV-7 represents a multifunctional protein which is not only engaged in TLS and cell cycle regulation as it was believed in the past, but also in many other significant biological processes and cancer-promoting conditions. They highlight that it remains unclear whether REV-7, which is involved in mutagenic TLS, contributes to cancer development by the introduction of de novo mutations [20]. Nevertheless, they did not analyze the immunohistochemical expression of REV-7 in human cancer tissues and its association with various clinicopathologic and prognostic factors. To the best of our knowledge, this is the first review, which systematically summarizes all existing evidence regarding immunohistochemical staining for REV-7 protein in pathology specimens and human tissues, originating from several different cancers.

Interestingly, in the aforementioned review by Murakumo et al., the authors stated that the understanding of the mechanisms which control REV-7 function in vivo will result in the development of effective molecular targeted therapy, by blocking REV-7 expression. Moreover, they summarized all recently developed REV-7 inhibitors [20]. Many of the articles included in our systematic review report the potential therapeutic utility of REV-7 protein and its role in cancer management and treatment. Feng et al. showed that inactivation of REV-7 in breast cancer cells resulted in reduced migration, invasion and epithelial-mesenchymal transition (EMT) of cancerous cells, while on the contrary, overexpression of REV-7 was associated with increased migration, invasion and EMT of breast cancer cells. They concluded that targeting REV-7 could represent a novel strategy for treating and preventing breast cancer [17]. Likewise, Sanoyama et al. showed that REV-7 plays an important role in small-cell lung cancer cell survival and proliferation, once again emphasizing the potential therapeutic properties of REV-7 inactivation, possibly through blockage of the TLS pathway, in esophageal SCCs [18]. On the contrary, Li et al. showed that the inactivation of REV-7 in colorectal cancer cells was associated with increased proliferation, colony formation and migration of cancerous cells, while overexpression of REV-7 resulted in the opposite effects. They concluded that modulations of the REV-7 protein could potentially represent a new therapy for colorectal cancer [15]. Finally, although Okina et al. also stated that inhibition of REV-7 function might become a therapeutic approach for patients diagnosed with diffuse large B-cell lymphoma who are resistant to rituximab-combined chemotherapy, they did not perform any experiments in order to confirm their hypothesis [16]. These contradictory results highlight the multifactorial role of this small protein in cancer and its differential effect according to cancer type.

In certain studies, chemosensitivity or radiosensitivity experiments were performed, by directly blocking REV-7. In particular, Sakurai et al. showed that the inactivation of REV-7 in testicular germ cell tumors increased chemosensitivity to cisplatin and doxorubicin, by promoting DSB accumulation and apoptotic pathways, while it also recovered chemosensitivity in cisplatin-resistant cancer cells. The authors concluded that REV-7 could represent an ideal molecular target for managing chemoresistant cases of testicular germ cell tumors [11]. Likewise, Niimi et al. showed that inactivation of REV-7 in clear cell carcinoma cells increased their sensitivity to cisplatin treatment. They thus arrived at the conclusion that immunohistochemical staining for REV-7 is a good tissue marker for predicting chemosensitivity, while at the same time, REV-7 protein represents a potential molecular target for the management of clear cell carcinomas [12]. Hoshino et al. showed that high REV-7 expression in malignant melanomas was associated with increased cancer cell proliferation, and that the inactivation of REV-7 in melanoma cells resulted in increased chemosensitivity to cisplatin but not to dacarbazine [19]. Finally, Gu et al. reported that high expression of REV-7 in esophageal SCCs was associated with radioresistance, both in vitro and in vivo. Furthermore, they stated that there is promising evidence that inactivation of REV-7 could result in increased radiosensitivity of SCCs, once again rendering REV-7 as a potential molecular target for cancer management and treatment [13]. Table 2 summarizes the potential therapeutic utility of REV-7 presented in selected studies.

**Table 2:** Potential therapeutic utility of REV-7 presented in selected studies.

Study Name	Target Tissue	Inactivation of REV-7	Target Therapy Potentials
Feng et al. [17]	Breast cancer	Reduced migration, invasion and epithelial-mesenchymal transition (EMT) of cancerous cells.	Yes
Sanoyama et al. [18]	Lung cancer	Significantly inhibited cell proliferation and survival.	Yes
Li et al. [15]​​​​​​​	Colorectal cancer	Increased proliferation, colony formation and migration of cancerous cells.	Yes
Okina et al. [16]​​​​​​​	Diffuse large B-cell lymphoma	The authors did not perform any experiments in order to confirm their hypothesis.	Yes
Sakurai et al. [11]​​​​​​​	Testicular cancer	Increased chemosensitivity to cisplatin and doxorubicin and recovered chemosensitivity in cisplatin-resistant cancer cells.	Yes
Niimi et al. [12]​​​​​​​	Ovarian cancer	Increased sensitivity of clear cell carcinoma cells to cisplatin treatment.	Yes
Hoshino et al. [19]​​​​​​​	Melanoma	Resulted in increased chemosensitivity to cisplatin but not to dacarbazine.	Yes
Gu et al. [13]​​​​​​​	Squamous cell esophageal cancer	Promising evidence that it could result in increased radiosensitivity of squamous cell carcinomas.	Yes

This review is not without limitations. To begin with, the number of included articles was small (n=9), while all selected studies were retrospective non-comparative ones. Moreover, data presented in selected studies were heterogeneous, and thus a statistical analysis was not feasible. All included articles, reported REV-7 immunohistochemical staining results in different cancer types and thus safe conclusions about the association between REV-7 expression and certain types of cancer cannot be safely drawn. Finally, included studies used different REV-7 antibodies and immunohistochemistry procedures and conditions, while immunohistochemistry evaluation and IS were performed by different reviewers.

## Conclusions

Positive REV-7 immunohistochemical expression was reported in several different types of cancer on human tissues, including testicular, prostate, ovarian, breast, esophageal, colorectal, lung and skin cancer and lymphomas. High REV-7 immunochemical expression was associated with faster disease progression, resistance to available management options and worse prognosis in the majority of cancers which were included in this systematic review. These results indicate that immunohistochemical staining of REV-7 protein could potentially be used as a predictive tissue marker in certain cases. Promising results, arising from REV-7 inactivation experiments, render REV-7 targeting a potential therapeutic strategy for future cancer management, especially in cases of chemoresistant or radioresistant disease. Further studies are needed in order to draw safer conclusions.
